# Introducing the pictogram-based ocular motor and visual-perceptual symptom scale: a multinational, cross-cultural feasibility study

**DOI:** 10.3389/fneur.2025.1636002

**Published:** 2025-07-29

**Authors:** Ali A. Melliti, Raymond Van de Berg, Evangelos Anagnostou, Ondrej Cakrt, Christian Chabbert, Wolfgang Heide, Christoph Helmchen, Jaroslav Jerabek, Erna Kentala, Hassen Kerkeni, Nehzat Koohi, Christophe Lopez, Leonel Luis, Dara Meldrum, Dusan Pavlovic, Maritta Spiegelberg, Luc Vereeck, Eva Grill, Klaus Jahn, Maja Striteska, Sophie Vanbelle, Andreas Zwergal, Johannes Gerb

**Affiliations:** ^1^Division of Balance Disorders, Department of Otorhinolaryngology and Head and Neck Surgery, Maastricht University Medical Centre, Maastricht, Netherlands; ^2^Department of Neurology, Eginition Hospital, National and Kapodistrian University of Athens, Athens, Greece; ^3^Department of Rehabilitation and Sports Medicine, 2nd Faculty of Medicine, Charles University and Motol University Hospital, Prague, Czechia; ^4^Aix Marseille Univ, CNRS, Centre de Recherche en Psychologie et Neurosciences (CRPN), Marseille, France; ^5^Department of Neurology, General Hospital Celle, Celle, Germany; ^6^Department of Neurology, University Hospital Schleswig-Holstein, University of Lübeck, Lübeck, Germany; ^7^Department of Otorhinolaryngology, University of Helsinki and Helsinki University Hospital, Helsinki, Finland; ^8^Department of Neurology, Inselspital, Bern University Hospital, University of Bern, Bern, Switzerland; ^9^Department of Clinical and Movement Neurosciences, Queen Square Institute of Neurology, University College London, London, United Kingdom; ^10^Department of ENT, Lisbon Academic Medical Center, Lisbon, Portugal; ^11^Academic Unit of Neurology, Trinity College Dublin, Dublin, Ireland; ^12^Hearing and Balance Centre Belgrade, Belgrade, Serbia; ^13^Department of Neurology, Cantonal Hospital of Baden, Baden, Switzerland; ^14^Department of Rehabilitation Sciences and Physiotherapy/Movant, Faculty of Medicine and Health Sciences, University of Antwerp, Antwerp, Belgium; ^15^German Center for Vertigo and Balance Disorders, LMU University Hospital, LMU Munich, Munich, Germany; ^16^Faculty of Medicine, Institute of Medical Data Processing, Biometry and Epidemiology, LMU Munich, Munich, Germany; ^17^Department of Otorhinolaryngology and Head and Neck Surgery, Faculty of Medicine in Hradec Kralove, University Hospital Hradec Kralove, Charles University, Hradec Kralove, Czechia; ^18^Department of Otorhinolaryngology, Third Faculty of Medicine, Charles University and University Hospital Kralovske Vinohrady, Prague, Czechia; ^19^Methodology and Statistics, CAPHRI, Maastricht University, Maastricht, Netherlands; ^20^Department of Neurology, LMU University Hospital, LMU Munich, Munich, Germany

**Keywords:** oscillopsia, vertigo, visual disorder, nystagmus, diplopia, health communication, neuro-otology

## Abstract

**Background:**

Patients with vestibular and ocular motor disorders often perceive oscillopsia, diplopia or visual hallucinations as their chief complaint. However, they often struggle with verbalizing these subjective ocular motor and visual-perceptual signs precisely, which complicates a correct diagnostic classification of the suspected pathogenic mechanism.

**Methods:**

In this multinational and cross-cultural feasibility study, a novel pictogram-based scale of 10 common ocular motor and visual-perceptual symptoms (called Pictogram Ocular Motor and Visual-Perceptual Symptom Scale, POVSS) was developed and validated. Healthcare professionals with or without expertise in neuro-ophthalmology and neuro-otology, representing a broad range of nationality and primary languages, were asked to match pictograms with medical symptoms (specialists) or a simple English symptom description (non-specialists).

**Results:**

A total of 174 participants (112 specialists, 62 non-specialists) from 30 nationalities evaluated the POVSS. On average, specialists reached a score of 9.7 out of 10 (SD = 0.5; 95% CI: 9.6–9.8) in matching symptoms and pictograms. Non-specialists achieved a mean score of 7.9 (SD = 2.3; 95% CI: 7.3–8.5) in accurately matching pictograms to simple English descriptions. In the specialist group, all pictograms met the common ISO quality standards, whereas in the non-specialist group, 8 out of 10 met the standards. While a significant difference in performance was found between the two groups, success rates did not differ between male and female participants.

**Conclusion:**

Visual-perceptual symptoms originating from common vestibular and ocular motor disorders could be reliably identified using the POVSS by healthcare professionals, independent of participant nationality, or gender. Further research is needed to test the clinical applicability of the POVSS in different patient care settings.

## Introduction

Disorders of the peripheral or central vestibular or ocular motor system, as well as the higher visual system can cause striking visually perceived disturbances such as oscillopsia, double vision, or scotoma (1). These symptoms were already described in ancient times, underlining their impact on patients’ wellbeing (2). While a precise evaluation of the patient complaints and case history is crucial for an accurate differential diagnosis in vestibular and ocular motor disorders (3, 4), discrepancies often exist between how patients describe their symptoms and how healthcare providers interpret them (5, 6). In consequence, the quality of the chief complaint, such as direction-specific turning or undirected swaying, has been described to be not sufficiently satisfactory in current diagnostic algorithms for patients with vestibular and ocular motor disorders (7, 8).

Effective communication between patients and healthcare providers is a cornerstone of quality medical care. Miscommunication can occur due to a variety of factors, including language barriers (9), cognitive impairments, emotional distress, or a lack of health literacy (10). This can lead to delayed diagnosis, diagnostic errors, and inadequate treatments (11). Moreover, patients from marginalized or underserved populations may face additional hurdles, such as cultural stigmas around expressing neurological symptoms or medication intake, which can further complicate their ability to articulate their concerns (12–14).

To ensure inclusivity and equity in neuro-ophthalmology and neuro-otology, it is essential to develop systems and practices that accommodate patients who might struggle with the verbal expression of ocular motor and visual-perceptual symptoms, while also providing physicians with tools to objectify patient-described subjective symptoms. In neuro-ophthalmological and neuro-otological conditions, misdiagnosis rates can reach up to 70% (15), potentially causing delay in patients care and management or even substantial harm (16). Different approaches for reducing misdiagnosis in the care of patients with vestibular and ocular motor disorders have been proposed (3, 17). In this clinical setting, in contrast to other fields of medicine (18–20), no visual aid was developed or tested so far. Thus, pictorial representations of visual disturbances, such as visual aura and oscillopsia, may improve the diagnostic process and enhance efficiency.

In this study, we aimed to assess the cross-cultural comparability of a novel, pictogram-based chart of 10 common visual-perceptual sensations. For this, members from the DIZZYNET, a trans-European network of vestibular researchers (21), and other international societies for neuro-otology, neuro-ophthalmology or ENT were contacted. The goal of this current study was to cross-culturally validate a language-independent scale for the understanding of common visual-perceptual symptoms related to ocular motor signs.

## Methods

### Questionnaire development

**Figure 1:**
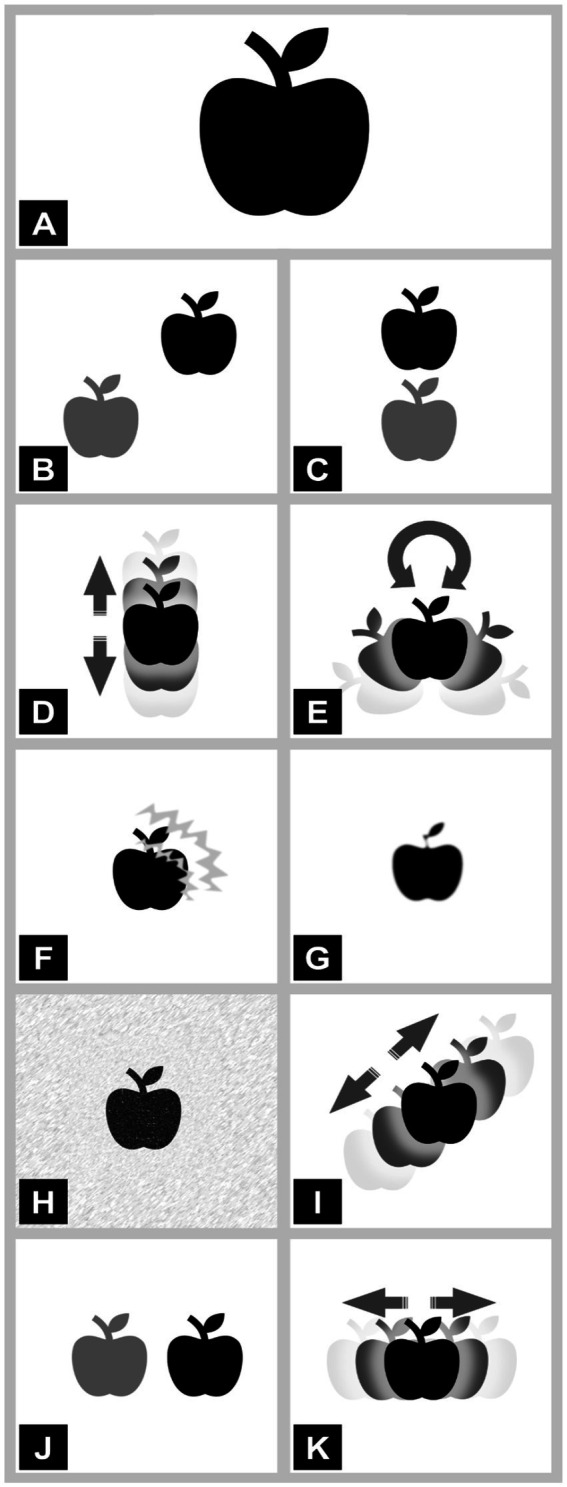
Pictograms of different visual disturbances: **(A)** reference, **(B)** diagonal double vision, **(C)** vertical double vision, **(D)** vertical oscillopsia, **(E)** torsional oscillopsia, **(F)** (migrainous) visual aura with fortifications, **(G)** blurred vision, **(H)** visual snow, **(I)** diagonal oscillopsia, **(J)** horizontal double vision, **(K)** horizontal oscillopsia.

A first draft of the Pictogram Ocular Motor and Visual-Perceptual Symptom Scale (POVSS) was created by two of the authors (AM, JG). Based on feedback during the 2024 annual DIZZYNET meeting, the draft was refined. The revised POVSS includes 10 pictograms for oscillopsia (vertical, horizontal, diagonal, torsional), double vision (vertical, horizontal, and diagonal), visual snow, blurred vision, and (migrainous) visual aura with fortifications (Figure 1). All pictograms are based on a picture of a black-and-white apple, which was then modified to symbolize the aforementioned visual disturbances. The pictograms utilize high-contrast in order to be easily readable. Movement is depicted by arrows, while double vision is symbolized by two objects (one in fainter color). This approach was chosen to be inclusive of all patient groups.

A survey consisting of 10 items was created, with each item featuring one pictogram accompanied by 10 multiple-choice answer options. All participants in the survey were asked to match the text that best described each pictogram and provide an answer for every question; skipping a question was not possible. The pictogram representing the absence of visual disturbance was provided as a reference in the beginning of the survey. Two versions of the survey, featuring the same pictograms with different text descriptions, were provided. One version was intended for participants with professional expertise in neuro-ophthalmology and neuro-otology (Specialist), while the other was designed for participants without such expertise (Non-specialist). Table 1 indicates the different terminology or descriptions used for each population. Both the order of the items and the response options within each item were randomized for each participant to minimize order effects and response bias.

**Table 1:** Description provided to the participants.

Specialist	Non-specialist
Diagonal double vision	Your surroundings appear to be doubled, with one copy being shifted diagonally
Vertical double vision	Your surroundings appear to be doubled, with one copy stacked above or below the other
Vertical oscillopsia	Your surroundings appear to be moving up and down
Torsional oscillopsia	Your surroundings appear to be moving in a circle
(Migrainous) visual aura	You see curvy or zigzag lines and triangles in one part of your vision
Blurred vision	Everything appears blurry and out of focus
Visual snow	You have the sensation of falling snow in your field of vision, no matter where you look
Diagonal oscillopsia	Your surroundings appear to be moving diagonally
Horizontal double vision	Your surroundings appear to be doubled, with one copy shifted horizontally, i.e., to the left or to the right of the other one
Horizontal oscillopsia	Your surroundings appear to be moving left and right

### Participant recruitment

Two of the authors (AM, JG) created the online questionnaire using the Qualtrics software. The link to the online survey was provided to DIZZYNET members as well as to various international neuro-ophthalmology, neuro-otology or ENT societies. Both neuro-ophthalmology/−otology specialists and non-specialists, including members of the general population, were included in the study. Exclusion criteria included age below 18 years, insufficient English language skills or active ocular motor, vestibular, visual, or central nervous disorder, which might affect visual perception. All participants were asked to provide their gender, primary language, current place of residence and profession. All data was collected anonymously. For privacy reasons, we chose not to ask participants’ age. This decision was made not to allow for immediate identification of participating DIZZYNET members.

### Setting and institutional review board approval

Approval was obtained from the ethical committee of Maastricht University Medical Center on December 6th 2024, in The Netherlands (METC 2024–0462). Informed consent was obtained from all participants. This study was conducted in accordance with the legislation and ethical standards on human experimentation in the Netherlands and in accordance with the Declaration of Helsinki (amended version 2013).

### Data analysis

Descriptive analyses were performed, summarizing quantitative variables as mean and standard deviation and qualitative variables as proportions. The correct classification of each pictogram was compared between specialists and non-specialists with a Chi-Square test when the expected frequency in each cell of the 2 × 2 contingency table was at least 5, and with a Fisher’s Exact test otherwise. To correct for multiple comparisons across the 10 individual pictograms, the significance threshold (*α*) was adjusted using a Bonferroni correction, resulting in a corrected alpha level of 0.005 (α = 0.05/10). The number of correct matches (treated as quantitative) was compared between specialists and non-specialists and between women and men, in both levels of specialization using independent samples *t*-tests. To account for multiple comparisons in the gender-based analyses, a correction was applied, adjusting the alpha level to 0.025 (α = 0.05/2).

The minimum comprehension threshold for pictograms (i.e., the rate of correctly identified pictograms) is 67% according to ISO (International Organization for Standardization) 9,186–1:2014, and 85% according to ANSI (American National Standards Institute) Z535.1–2006 (R2011) (22). These thresholds were used as reference criteria for evaluating pictogram comprehension in this study. All data was anonymously processed using the Qualtrics software (Qualtrics, Provo, UT, United States), SPSS v28.0.0.0 (IBM, Armonk, New York, United States), Microsoft Excel 2016 (Microsoft, Redmond, Washington, United States), and JASP.^1^

## Results

### Participant demographics

A total of 179 responses were collected; after excluding five participants (four declined consent, one gave uniform responses indicating a lack of engagement or trouble understanding the instructions), 174 valid responses remained. Participants represented 30 nationalities (Supplementary material), 17 primary languages, and 24 countries of residence. Of these, 112 were self-identified specialists (64 female/46 male/2 prefer not say) and 62 (40 female/22 male) were non-specialists. Detailed participant demographics, including nationality, language, and profession, are provided in Table 2.

**Table 2:** Demographic data, self-assessed ethnicities and nationalities (sorted alphabetically) by all participants.

Characteristics	Count (*n* = 174)
Gender	
Female	104 (59.8%)
Male	68 (39.1%)
Prefer not to say	2 (1.1%)
Primary language	
Arabic	22 (12.6%)
Armenian	1 (0.6%)
Chinese	3 (1.7%)
Czech	1 (0.6%)
Dutch	15 (8.6%)
English	13 (7.5%)
French	95 (54.6%)
German	8 (4.6%)
Greek	3 (1.7%)
Italian	2 (1.1%)
Luxembourgish	1 (0.6%)
Malayalam	2 (1.1%)
Nepali	1 (0.6%)
Romanian	4 (2.3%)
Spanish	1 (0.6%)
Turkish	1 (0.6%)
Vietnamese	1 (0.6%)
Expertise	
Specialist	112 (64.4%)
Non-specialist	62 (35.6%)
Profession	
Audiologist	31 (17.8%)
Audiovestibular physician	5 (2.9%)
ENT	33 (19%)
Medical assistant	1 (0.6%)
Neurologist	13 (7.5%)
Neurophysiologist	1 (0.6%)
Neurophysiology, neuro-otology PhD student	1 (0.6%)
Non-medical professional	10 (5.7%)
Nurse	1 (0.6%)
Ophthalmologist	1 (0.6%)
Orthoptist	45 (25.9%)
Physical therapist	30 (17.2%)
Psychomotor therapist	1 (0.6%)
Researcher in neurosciences	1 (0.6%)

### Accuracy analysis

**Figure 2:**
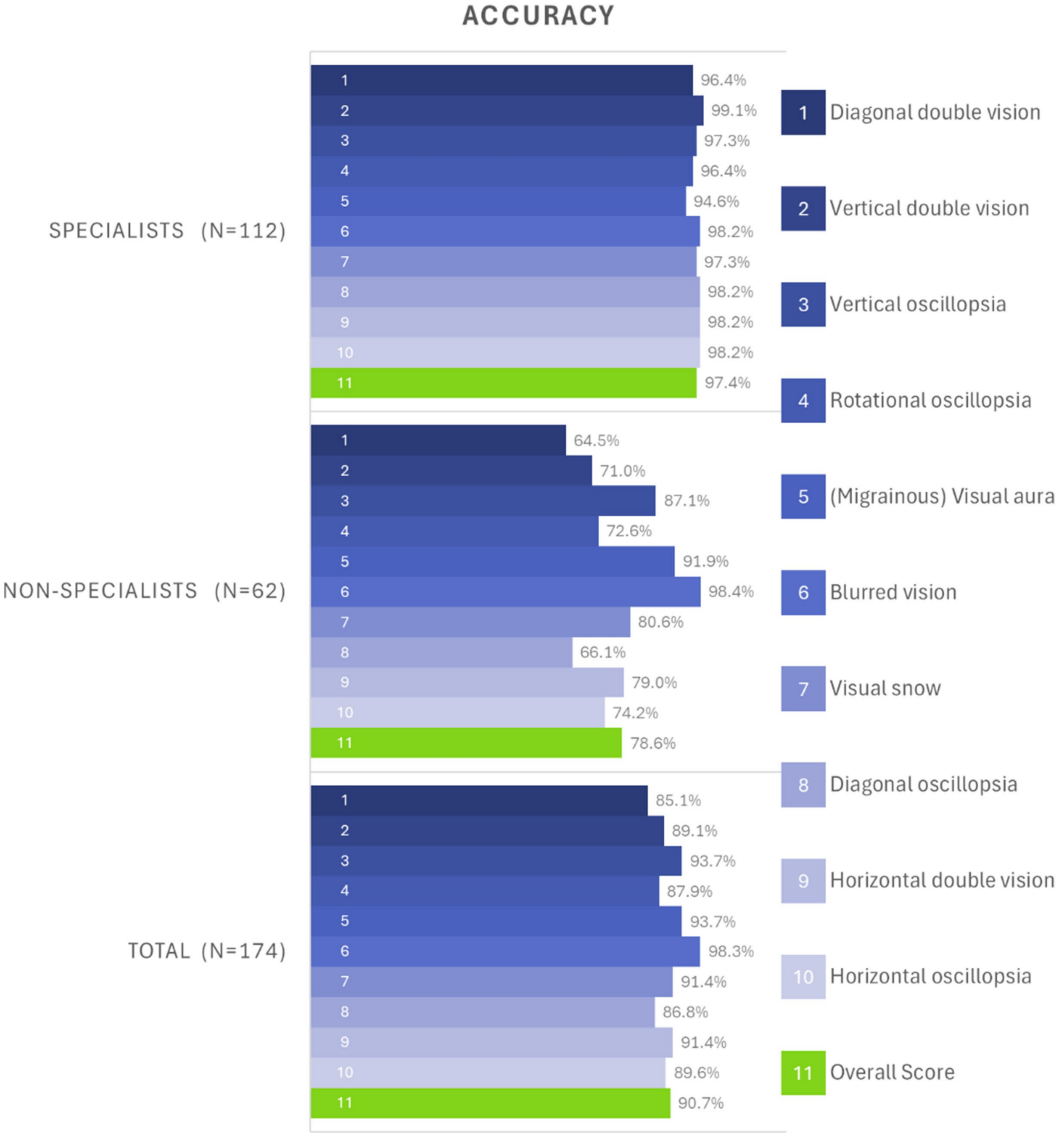
Bar chart of overall accuracy rates per pictogram, divided by specialists (top), non-specialists (center), and grouped from all participants (bottom). The 10 different test items are depicted in shades of blue, and the overall average score is depicted in green. For example, test item 3 (“vertical oscillopsia”) was correctly identified by 97.3% of specialists (third row of top chart), and by 87.1% of non-specialists (third row of center chart). Note that the non-specialists were given a simple English description of the symptom (“Your surroundings appear to be moving up and down” instead of “vertical oscillopsia”). While some pictograms were easily understandable by non-specialists (e.g., “(Migrainous) Visual aura” and “Blurred vision”), others were difficult to tell apart (e.g., “Diagonal oscillopsia” and “Diagonal double vision”) for the non-specialists.

On average, the specialists demonstrated a mean score of accuracy of 9.7 out of 10 (SD = 0.5, 95% CI: 9.6–9.8) in correctly identifying the pictograms. The non-specialists showed a mean score of 7.9 (SD = 2.3, 95% CI: 7.3–8.5) in accurately matching pictograms to simple English descriptions. In all but three pictograms, the specialists significantly outperformed the non-specialists. All pictograms met the minimum comprehension thresholds (i.e., the rate of correctly identified pictograms) established by both ISO (67%) and ANSI (85%) standards for specialists. However, among non-specialists, two pictograms did not meet either criterion, five met only the ISO criterion, and three met both criteria. Detailed success rates per pictogram can be seen in Table 3 and Figure 2.

**Table 3:** Number of participants accurately matching pictograms to provided descriptions (accuracy rate).

Visual disturbance	Specialists agreement (*n* = 112)	Non-specialist agreement (*n* = 62)	*p*-value	Total (*n* = 174)
Diagonal double vision	108 (96.4%)^†^	40 (64.5%)	<0.001*	148 (85.1%)^†^
Vertical double vision	111 (99.1%)^†^	44 (71.0%)^‡^	<0.001*	155 (89.1%)^†^
Vertical oscillopsia	109 (97.3%)^†^	54 (87.1%)^†^	0.018	163 (93.7%)^†^
Torsional oscillopsia	108 (96.4%)^†^	45 (72.6%)^‡^	<0.001*	153 (87.9%)^†^
(Migrainous) Visual aura	106 (94.6%)^†^	57 (91.9%)^†^	0.524	163 (93.7%)^†^
Blurred vision	110 (98.2%)^†^	61 (98.4%)^†^	1.000	171 (98.3%)^†^
Visual snow	109 (97.3%)^†^	50 (80.6%)^‡^	<0.001*	159 (91.4%)^†^
Diagonal oscillopsia	110 (98.2%)^†^	41 (66.1%)	<0.001*	151 (86.8%)^†^
Horizontal double vision	110 (98.2%)^†^	49 (79.0%)^‡^	<0.001*	159 (91.4%)^†^
Horizontal oscillopsia	110 (98.2%)^†^	46 (74.2%)^‡^	<0.001*	156 (89.7%)^†^
Mean of overall score (out of 10)	9.7 (SD = 0.5; 95% CI: 9.6–9.8)	7.9 (SD = 2.3; 95% CI: 7.3–8.5)	<0.001*	9.1 (SD = 1.7; 95% CI: 8.9–9.4)

^†^Meets both ISO (≥67%) and ANSI (≥85%) criteria. ^‡^Meets only ISO (≥67%) criteria. *Significant effect of the group.

The specialists significantly outperformed the non-specialists in the overall score. The participant gender did not constitute a significant factor for the overall success rate in either the specialist or non-specialist populations (independent samples *t*-test: n.s.).

**Figure 3:**
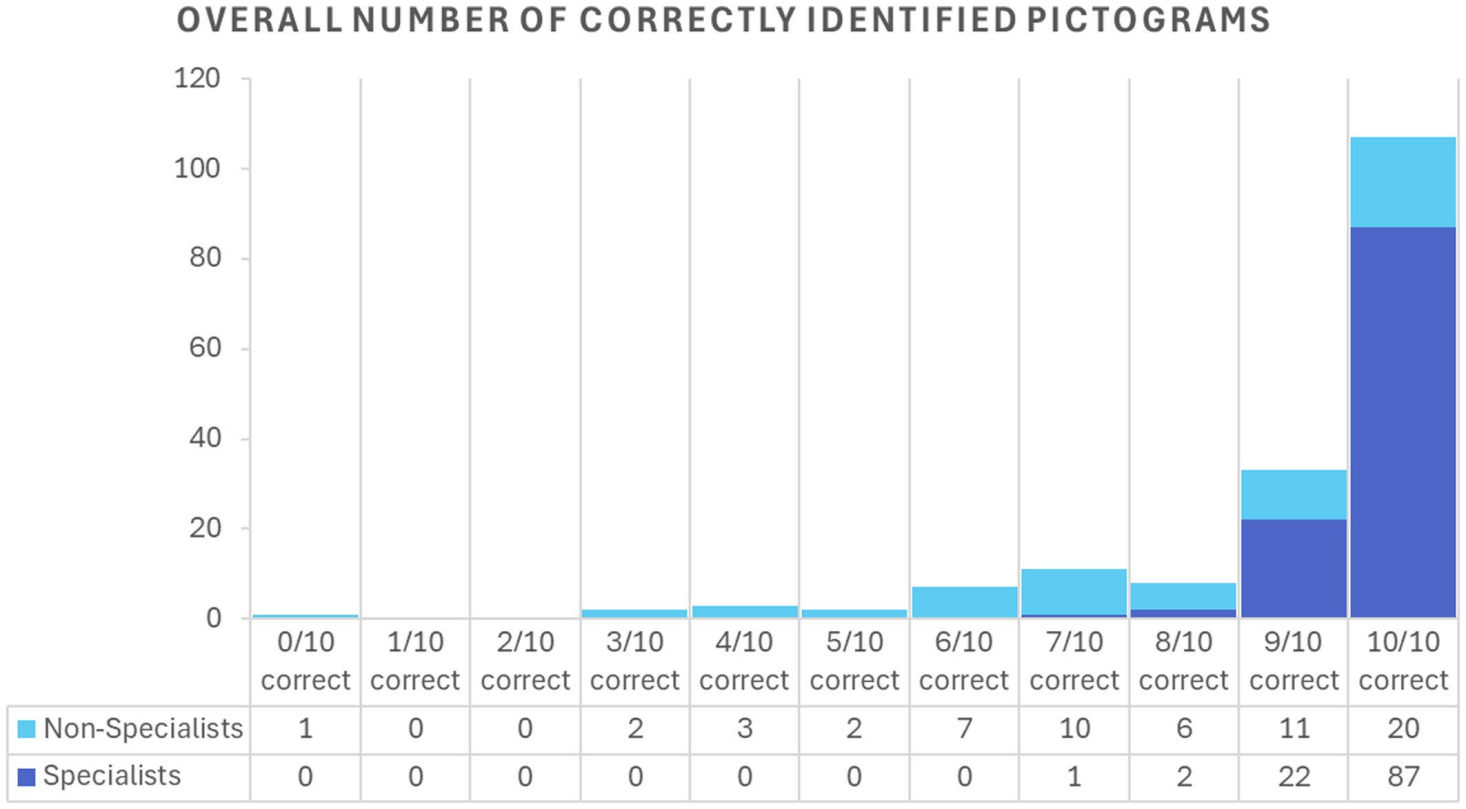
Distribution of the overall number of correctly identified pictograms per expertise level.

Among specialists, the mean score for female participants was 9.8 out of 10 (SD = 0.5; 95% CI: 9.7–9.9), and for male participants, 9.6 (SD = 0.6; 95% CI: 9.4–9.8) (*p* = 0.086). Similarly, among non-specialists, female participants showed a mean score of 7.7 (SD = 2.0; 95% CI: 7.1–8.3), and male participants 8.1 out of 10 (SD = 2.7; 95% CI: 7.0–9.2) (*p* = 0.47). The two participants who chose “prefer not to say” were excluded from the gender-based analysis. Figure 2 shows the accuracy for each pictogram, globally and per specialty while Figure 3 shows the distribution of rate of correctly identified pictograms per participant (0–10), grouped by expertise level (specialists vs. non-specialists).

## Discussion

In this first feasibility study, participants with and without prior expertise in diagnosing neuro-ophthalmological and neuro-otological disorders showed good to excellent accuracy in correctly matching pictograms of common visual-perceptual disturbances to their corresponding medical symptoms. The pictograms effectively conveyed their intended meaning, regardless of participant’s native language or gender.

Overall, specialists demonstrated a near-perfect accuracy rate of 97% in identifying medical symptoms using pictograms. In contrast, the non-specialist group exhibited a significantly lower accuracy rate of 79%, which is still within the range considered as “good” accuracy (23). However, two pictograms did not meet the ISO criterion; both involve diagonal visual disturbances. This lower rate partially stems from the study design: non-specialists especially struggled in differentiating visualizations of oscillopsia and diplopia, which are naturally difficult to visualize using static pictograms only. Here, video depictions might result in higher accuracy rates. In a similar approach, Holly et al. could show how using video language can aid patients with BPPV to communicate their experiences (24). Another potential factor could lie in the text descriptions of each symptom, which might have been difficult to understand for non-specialists. In future versions of the descriptions, the readability should therefore be improved, e.g., by rephrasing the descriptions for diagonal oscillopsia and diagonal double vision, which were the most problematic. Furthermore, for people unfamiliar with ocular motor disorders, the difference between oscillopsia and diplopia might be difficult to grasp without further explanation. This, however, might not be the case for actual patients experiencing these symptoms, who might solely struggle to exactly verbalize their individual complaints. Especially in cases of complex symptomatology and long patient journeys, patients tend to gradually become better at communicating their symptoms (25). POVSS might therefore be useful as a fast, visual aid for clinicians, helping patients to express their subjective complaints. It should be noted that POVSS is, however, not meant as a standalone, unsupervised diagnostic tool.

There are several limitations to this study. Firstly, participants were predominantly European, despite efforts to contact societies in various countries. While the sample still remains relatively diverse, encompassing 30 different nationalities and 17 distinct primary languages, roughly half of the participants were French. Follow-up investigations might be needed to ensure POVSS understandability in currently underrepresented countries or nationalities.

Secondly, only English-speaking participants were recruited. Although the inclusion criteria required a proficient level of English, participants’ language skills were not formally assessed. This lack of assessment may have influenced the accuracy of the responses, particularly in the non-specialist cohort. Additionally, the descriptions provided to specialists and non-specialists differed. The descriptions used for specialists included medical terminology, whereas those for non-specialists used non-medical language. These descriptions were developed by medical experts based on their experience with patients, and are currently only available in English language. Before conducting future studies in an international patient cohort, a cross-cultural translation and validation (26) of the item descriptions is therefore needed.

In addition, the purpose of this study was to determine whether the pictograms effectively convey their intended meaning before proceeding with clinical validation. Therefore, no patients were recruited. Research in clinical settings is mandatory to test the applicability and usefulness of the scale. This might be mostly relevant in an emergency care setting, where vestibular symptoms (and associated ocular motor and visual signs) are common, yet difficult, complaints to stratify (8, 27). Here, multiple potentials pitfalls need to be considered: for example, patients with cognitive impairment or patients with lower formal education levels might struggle to understand the test instructions, especially in an unsupervised implementation of POVSS. Furthermore, patients with severe visual impairment due to an ophthalmologic disorder (e.g., glaucoma), might find it hard to use the scale. Another potential limitation might be due to spatial orientation deficits, which are a common part of vestibular disorders (28, 29): due to spatial disorientation, patients might find it difficult to clearly indicate the direction of a visual disturbance. Future studies in patients are therefore needed. These could investigate both the diagnostic value of POVSS, i.e., from the clinicians’ perspective, as well as the perceived simplification of symptom communication from the patients’ perspective.

In conclusion, this feasibility study, based on evaluations from both specialists and non-specialists, demonstrated that the pictograms effectively conveyed common ocular motor and visual-perceptual symptoms. The next step will be to assess the validity of these pictograms in real-world clinical settings with patients who actually experience such symptoms.

## Data Availability

The raw data supporting the conclusions of this article will be made available by the authors, without undue reservation.

## Footnotes

^1^ jasp-stats.org
