# Supplementary material for: Introducing the pictogram-based ocular motor and visual-perceptual symptom scale: a multinational, cross-cultural feasibility study
Source: Front Neurol. 2025 Jul 29;16:1636002. doi: 10.3389/fneur.2025.1636002 (PMC12340241; doi:10.3389/fneur.2025.1636002)
Supplement: Supplementary file 1 [file Table_1.DOCX]

**Supplementary Material**

| **Country** | **Count(n=174)** |
| --- | --- |
| Algeria | 1 (0.6%) |
| Armenia | 2 (1.1%) |
| Australia | 1 (0.6%) |
| Bahamas | 1 (0.6%) |
| Belgium | 14 (8%) |
| Canada | 1 (0.6%) |
| Chile | 1 (0.6%) |
| China | 3 (1.7%) |
| Czech Republic | 1 (0.6%) |
| Egypt | 1 (0.6%) |
| France | 88 (50.6%) |
| Germany | 6 (3.4%) |
| Greece | 3 (1.7%) |
| India | 2 (1.1%) |
| Italy | 2 (1.1%) |
| Jordan | 2 (1.1%) |
| Lebanon | 1 (0.6%) |
| Luxembourg | 1 (0.6%) |
| Morocco | 2 (1.1%) |
| Nepal | 2 (1.1%) |
| Netherlands | 6 (3.4%) |
| Romania | 4 (2.3%) |
| Saudi Arabia | 2 (1.1%) |
| Switzerland | 2 (1.1%) |
| Syrian Arab Republic | 12 (6.9%) |
| Tunisia | 2 (1.1%) |
| Turkey | 1 (0.6%) |
| United Kingdom of Great Britain and Northern Ireland | 2 (1.1%) |
| United States of America | 7 (4%) |
| Vietnam | 1 (0.6%) |
